# Ageing, clinical complexity, and exercise therapy: a multidimensional approach

**DOI:** 10.3389/fspor.2024.1422222

**Published:** 2025-01-06

**Authors:** Salvatore Corrao, Dario Cerasola, Daniela Lucini, Christiano Argano

**Affiliations:** ^1^ Department of Health Promotion Sciences, Maternal and Infant Care, Internal Medicine and Medical Specialties, (PROMISE), University of Palermo, Palermo, Italy; ^2^ Department of Internal Medicine, National Relevance and High Specialization Hospital Trust, ARNAS Civico, Di Cristina, Benfratelli, Palermo, Italy; ^3^ Department of Psychology, Educational Science and Human Movement, University of Palermo, Palermo, Italy; ^4^ Department of Experimental Biomedicine and Clinical Neurosciences (BioNeC), University of Palermo, Palermo, Italy; ^5^ BIOMETRA Department, University of Milan, Milan, Italy; ^6^ Exercise Medicine Unit, Istituto Auxologico Italiano, IRCCS, Milan, Italy

**Keywords:** ageing, clinical complexity, exercise therapy, physical exercise, active ageing, multidimensional approach

## Abstract

Ageing is a multidimensional concept related to the progressive decline in physiological functions. The decrease of physical autonomy due to the ageing process leads to frailty, which in turn is associated with disability and comorbidity. Ageing represents the primary risk factor for chronic degenerative diseases, especially involving cardiovascular, metabolic, respiratory, and osteoarticular systems, determining the decrease in activities and quality of daily life. Regular physical activity can significantly reduce the risk of chronic degenerative diseases, moderate or severe functional limitations, and premature death in older adults. In light of the relationship between ageing, sedentary lifestyle, disability, comorbidity, and physical activity, a clear need emerges within the health system. Better control on the territory of pathologies related to ageing with the management of clinical and care complexity, multidimensional and multi-professional evaluation of the elderly complex and fragile patient, also through the definition of specific outpatient packages for pathology that allow the simplification of the evaluation process. There is a need for integration between local services, hospitals, and social assistance services. Aim of this review is to highlight the importance of the multidimensional approach is essential to be able to classify the complex elderly patient according to a multi-professional vision aimed at evaluating comorbidities and frailties, including sarcopenia, nutritional deficits, functional capacity, and planning a personalized and monitored motor training program, to improve motor, metabolic, cardiovascular and respiratory functions. In addition, developing an integrated hospital-community-care services management program to follow up with patients post-care is crucial.

## Introduction

In high-income countries, people are getting older. In Europe, the segment of people aged 65 and older is projected to increase from 14% in 2010 to 25% in 2050, making it the highest globally (1). According to the American College of Sports Medicine (ACSM) position (2), in the United States by 2030, the number of individuals who are 65 years old or older is projected to reach 70 million, and persons aged 85 years or older will be the fastest-growing part of the population (2).

Different measures, from lifestyle to medical care, including food quality and prevention improvements, have supported this trend. This tendency is more interested in healthy ageing. Since a more extended lifespan does not always reflect prolonged well-being, the primary efforts address disease avoidance and preservation of physical function into old age (3, 4).

Indeed, in older adults, the ability to perform daily tasks is measured by functional capacity, including grooming and other activities, dressing, feeding, and cooking, as well as more complex activities such as managing finances, driving, and using technology is becoming increasingly limited. This process, called “disability”, is defined as “difficulty or dependency in carrying out activities necessary for independent living, including roles, tasks needed for self-care and household chores, and other activities essential for a person's quality of life” (5), has been extensively studied. Self-reported questionnaires or performance-based tests diagnose disability as a medical condition. In particular, in persons aged 60 years and older, disability screening by evaluating risk factors such as impaired mobility, muscle strength, balance, and sensory functions are recommended (6, 7). In the elderly population, physical activity is decreased compared to young adults, as indicated by different methods such as self-report and interviews, body motion sensors, and estimates of daily caloric expenditure. It is well known that ageing is the major risk factor for many chronic diseases. The reason why a fundamental approach would be to step into the underlying ageing process (8). Ageing is a natural process characterized by the progressive degeneration of molecules, cells, and tissue, which hurts the structure and function of vital organs (9). The world's population is growing older, which poses challenge to society because, in the future, there will be a growing need for healthcare services for individuals with chronic conditions who are part of the more vulnerable population. The sedentary lifestyle contributes, along with other risk factors (e.g., alcohol, smoking, and irregular diet), to the development of numerous chronic degenerative diseases involving, in particular, cardiovascular, metabolic, and osteoarticular systems (10–12).

On the other hand, regular physical activity leads to significant health benefits in all age groups. Recent studies outline the favourable effects of physical activity on health, even during the COVID-19 pandemic (13–15). Physical activity is not contraindicated in old age; it can prevent cardiovascular diseases, morbidity, and disability of older adults (16). Generally, in the elderly, physical exercise or (defined as activity, structured, repetitive, and voluntary that improvement or maintenance of one or more components of physical fitness) or physical activity (defined as any energy expediture produced by muscles movement) can improve muscle tone and the ability to move, reduce osteoporosis, and induce an increased release of neurohormonal mediators such as endorphins and serotonin, giving a feeling of general well-being. Regular physical exercise or activity has different positive aspects (17). First, they reduces the risk of developing chronic conditions such as cardiovascular, metabolic, and tumoral diseases. Specifically, it decreases the risk of developing type 2 diabetes by 50%, the burden of hypertension, and osteoporosis (with a reduction of up to 50% of the risk of hip fracture in women) (17, 18).

Data from the literature showed that regular physical activity reduces the risk of developing cognitive impairment and dementia, a reduction of symptoms of anxiety, stress, depression, solitude, and weight loss, and decreases obesity risk compared with subjects with a sedentary lifestyle (19, 20).

However, it is essential to remember that the ageing process involves some inherent limitations to functional reserves. Regular physical activity can improve older people's functional capacity and quality of life. Given this background, this review aims to deepen the relationship between health, ageing, physical activity and physical exercise, establish a new healthcare model for avoiding hospitalization, and answer elderly healthcare needs. In addition, this review focus on the importance of the multidimensional approach as tool for a multi-professional vision aimed at evaluating comorbidities and frailties.

## Ageing and clinical complexity: comorbidity, frailty and disability

World life expectancy at birth has increased by about 30 months per decade since the middle of the 19th century (21). The leading risk factor for common chronic and killer conditions such as metabolic, cardiovascular, neurodegenerative disease, and malignancy is increasing age, which is the major problem of longer lives (22). By 2050, the number of people over 60 is expected to increase from 605 million in 2000 to 2.1 billion (23). Ageing is becoming a critical complexity concept for its public health consequences, such as adverse health outcomes and increased costs, as well as for the impaired quality of life of the older population (24).

Ageing is the natural decline in physiological function (25) due to the balance between injury and repair, cell death and cell replacement to maintain organ integrity (26).

**Figure 1:**
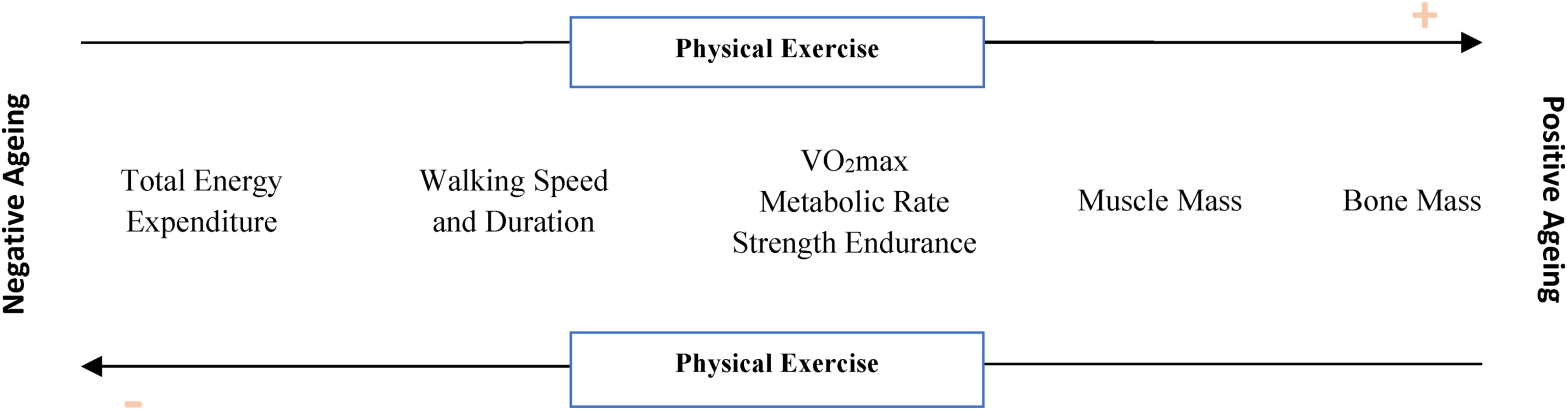
Physical exercise and related functional and structural changes: relationships with positive and negative ageing.

Notwithstanding, ageing is a multidimensional, nonlinear process associated with individual system changes and connections and interactions among system components (Figure 1). This process is characterized by progressive degeneration of tissues with a negative impact on the structure and function of vital organs. Cumulatively, it can influence daily life activities and the maintenance of physical autonomy in the older age group (8, 27). According to Dodd's (27) definition, there are three groups of physiological changes associated with age: (1) cellular homeostasis mechanisms (body temperature, blood and extracellular fluid volumes); (2) decrease in organ mass; (3) decline and loss of the functional reserve of the body.

In particular, the ageing process is not a disease itself but is one of the most critical risk factors for many chronic conditions, increasing vulnerability to disease (28). The principal area of change in the body that occurs with age is remarkably long and includes changes in the respiratory system, central nervous system, cardiovascular system, renal system, endocrine system, gastrointestinal system, and body and bone composition (Table 1).

**Table 1:** Summary of physiological changes of ageing.

Principal areas of changes with ageing	Changes with ageing	Clinical consequences
Central nervous system	Neuronal loss Lens opacifications Slowed reaction time Increase lens rigidity Dorsal column loss Anterior horn cell loss	Increase the risk of falls Reduce sense Increase risk of delirium Muscle weakness Presbyopia Cataract
Gastrointestinal system	Reduce motility	Constipation
Endocrine system	Deterioration in pancreatic β–cell function	Risk of impaired glucose tolerance
Cardiovascular system	Reduced maximum heart rate Dilatation of aorta Reduced elasticity of conduit/capacitance vessels Reduced number of pacing myocytes in the sinoatrial node	Reduce exercise tolerance Increase risk of postural hypotension Increase risk of atrial fibrillation Widened aortic arch on x—ray Widened pulse pressure
Respiratory system	Reduced lung elasticity and alveolar support Increased chest wall rigidity Increased V/Q mismatch Reduced cough and ciliary action	Reduced vital capacity and peak expiratory flow Increased residual volume Reduced inspiratory reserve volume Reduced arterial oxygen saturation Increased risk of infection
Bones	Reduced bone mineral density	Increased risk of osteoporosis
Renal system	Loss of nephrons Reduced glomerular filtration rate Reduced tubular function	Impaired fluid balance Increased risk of dehydration/overload Impaired drug metabolism and excretion

**Figure 2:**
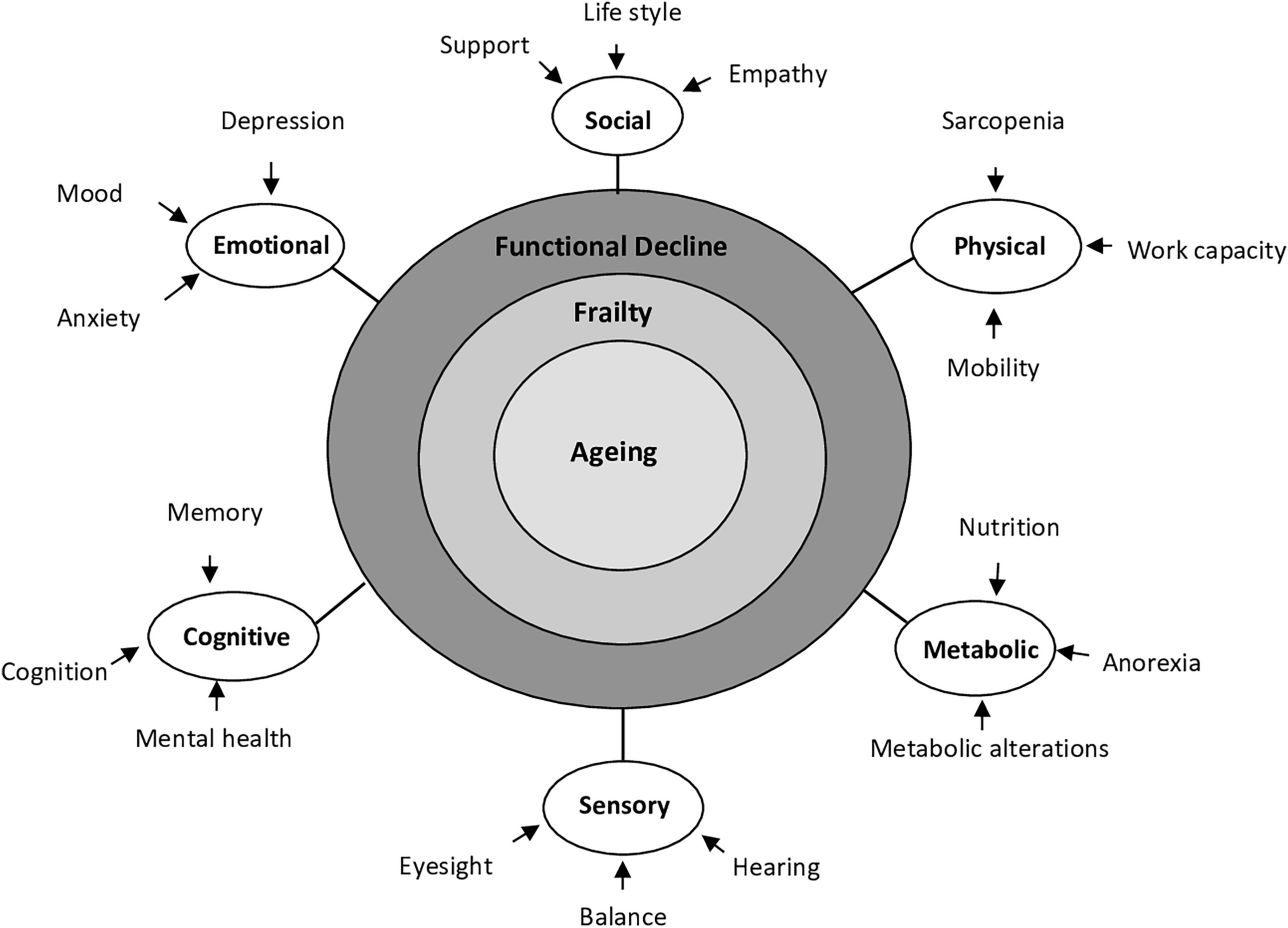
Graphic representation concept of association among factors ageing, frailty and decline. Model of ageing impairment characteristics that includes emotional, social, physical, metabolic, sensory and cognitive parameters.

The functional decline due to ageing leads to frailty (Figure 2). Frailty is linked to the term disability, comorbidity and advanced old age (29, 30). Frailty is the physiological decline in late life characterized by cumulative declines across multiple systems (5). Mainly, frail older adults are less able to adapt and withstand stress, such as acute illness or trauma. Frailty can stem from various factors such as social, psychological and physical factors, or a combination of these. It may involve a decline in muscle mass and strength, reduced energy levels, decreased physical exercise or physical activity tolerance, cognitive impairment, and a reduced physiological capacity. These factors can lead to poor health outcomes and a decreased ability to recover from acute stress. Regarding functional decline, three stages in the frailty process can be described: pre-frail, frailty state and complications (31). The first state, “pre-frail process”, clinically silent, is characterized by physiological reserves sufficient to allow the organism to respond adequately, with a complete recovery, to any acute disease, injury or stress.

The second state, “frailty”, refers to insufficient functional reserves that determine a slow and incomplete recovery after any new acute disease, injury or stress. The third condition, “complications of the frailty process,” is directly related to functional decline that results from impaired homeostatic reserve and reduced organismic capacity to withstand stress. This state leads to disability, comorbidity conditions, an increased risk of hospitalization, cross-infection and death (32, 33). Sometimes, with advancing age, the functional decline reaches the disability threshold. As above mentioned, disability is defined as difficulty in carrying out activities necessary for independent living. These include roles, tasks needed for self-care and household chores, and other activities essential for a person's quality of life (5, 34). Physical disability is not only associated with ageing but also with social, economic, and behavioural factors.

Another concept linked to ageing is comorbidity. Comorbidity is a simple concept: It is a term used to describe the presence of two or more medically diagnosed diseases in the same person. The diagnosis of each contributing disease is based on established, widely recognized criteria (35). Comorbidities not only affect the severity of symptoms and quality of life for each patient but also impact the prognosis, including the risk of hospitalization and death (36). In this regard, recent studies identified sex differences in disease distribution in hospitalized older people, highlighting a men's profile characterized by diabetes, chronic kidney disease, COPD, coronary artery disease, and malignancy and a women's profile characterized by hypertension, anaemia, osteoarthritis, depression, and diverticulitis disease (37). Frailty is distinct from comorbidity and disability but overlaps with both entities. Marcucci et al. identified four frailty phenotypes associated with different outcomes, pointing out the association between the phenotype linked to severe multimorbidity and higher in-hospital and 3-month mortality (38). Cesari et al. (39) suggested using the frailty index to weigh the risk profile of hospitalized patients (39).

Moreover, frailty and comorbidity predict disability, adjusting for each other; the disability may well exacerbate frailty and comorbidity, and comorbid diseases may contribute to the development of frailty. It is not uncommon for older adults to concurrently have comorbid disorders and be frail and disabled. Corrao et al. (40) showed that disability represents a strong predictor of mortality in older inpatients with pneumonia (40). However, comorbidity, frailty and disability do not entirely define the complexity of medical patients.

**Figure 3:**
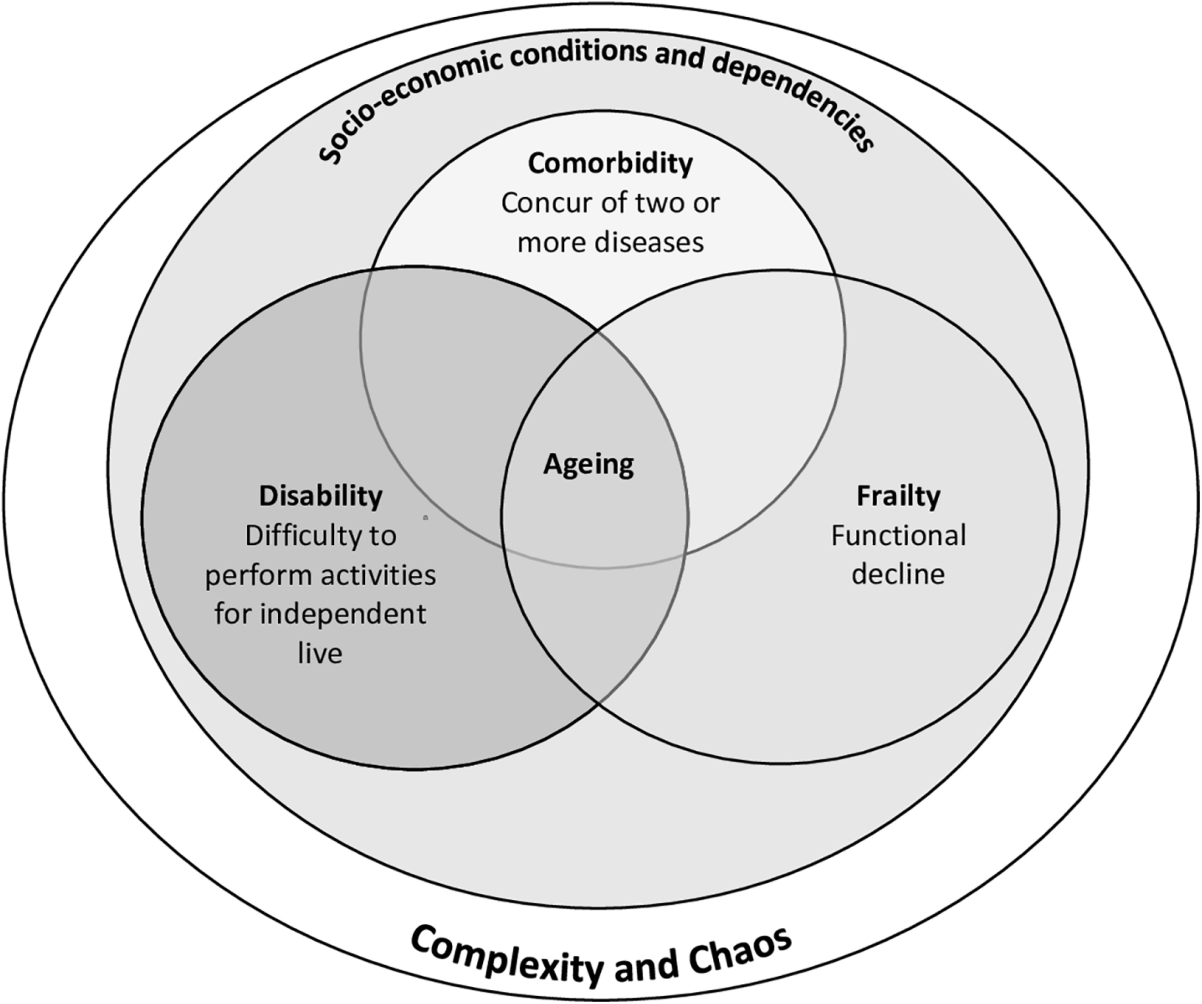
Critical elements that concur to clinical complexity or chaos. Association among frailty, comorbidity, disability and their relationship with socio-economic and dependencies factors.

The complexity of a system refers to its degree of complication (35, 41), and the clinical condition of an older adult can be explained by complex relationships between various factors (Figure 3). Recently, a new predictive model has been proposed to measure complexity: the FADOI-COMPLIMED scores. This model encompasses two scores: The first score measures the degree of dependence and frailty; it represents the first dimension of complexity, and the second score measures the degree of comorbidity indicated as the second dimension of complexity (42).

## Physical fitness and decline

The term “physical fitness” defined as the movements that people perform, in relation to vigor, alertness and ample energy to deal with the dynamics of everyday life. This definition includes sports and other activities such as playing, walking, dedicating to housework or gardening (43). The aging tends to reduce physical fitness with particular refer to aerobic and anaerobic physiological component, flexibilityand balance. In specific:

– Aerobic exercise refers to an effort that can be sustained continuously without interruption over a prolonged period of time (44), and is associated with endurance sport (marathon, cycling). The exercise activates specific muscle groups, which extract energy in the form of ATP from amino acids, carbohydrates and fatty acids (aerobic metabolism). According to Haskell et al. (43), aerobic exercises result from the cardiorespiratory system's ability to supply oxygen and the skeletal muscles' capacity to use oxygen. The parameter measure for the aerobic component is the maximum volume of oxygen consumed per minute (VO_2_ max), which is the product of maximal cardiac output and the maximal arteriovenous oxygen difference (44). It can be measured with a stress test by a graded exercise ergometry or treadmill protocols with an oxygen consumption analyzer or *via* mathematical formulas (43–46). It is important to note that direct measurement is considered to be the most accurate method for evaluating a person's aerobic power. A study by Buskirk et al. (47) demonstrated that VO_2_ max value decreases by approximately 1% per year in non-training individuals after age 25. As a result, the VO_2_ max of an untrained elderly person is considerably lower than that of an untrained young individual. However, this decline in maximum oxygen consumption is around 0.5% per year in master athletes who engage in aerobic activities (48). Three significant factors cause VO_2_ max decline in sedentary people: (1) a decline in muscle oxidative capacity due to ageing and/or inactivity (49), (2) a decline in maximal cardiac output (50), and (3) a decrease in muscle mass with a corresponding increase in fat mass, which is metabolically inactive (51). Aerobic exercise increases maximal oxygen uptake and stroke volume, maximal exercise times, and reduced heart rate and blood pressure values at rest and during submaximal exercise. It burns more calories than resistance training. It also contributes to more reductions in the percentage of body fat.
– Anaerobic exercise is defined as intense physical activity of a concise duration, fuelled by energy sources and produced by ATP creatine phosphate (CP) and glycolytic metabolism within the muscles, without using oxygen. It is associated with resistance training (43). In particular, this process produces significantly less ATP than its aerobic counterpart and leads to the build-up of lactic acid (52). The factors measured for the anaerobic component are defined as “the muscular strength power”. It can be measured by a 30 s wingate test with particular refer to measure two primary measures: anaerobic capacity and anaerobic power outputs (53).The ageing process causes a quantitative and quality loss of muscle mass (sarcopenia) and decreased strength and muscle power (54). The strength's peak occurs between the second and third decades of life. A slight decrease in muscle strength occurs up to 50 years, accentuated after 65, and then decreases from 12% to 15% each decade (55, 56).
– A joint's range of motion defines flexibility (54). Lack of flexibility may increase injury rate and cause functional problems, particularly in the sedentary, middle-aged, and elderly (57). Flexibility declines by 20 to 30% between the ages of 30 and 70 (58).
– Balance is the ability of a person to successfully maintain the position of their body, in particular, to maintain the centre of gravity within specific boundaries of space, and it can be either static (at rest) or dynamic (during motion) (59). Balance system functions include correcting for an accidental displacement of the centre of gravity, providing perceptual information about body positioning, and maintaining a clear image of the environment.In contrast, the body is in motion, respectively. Balance decline is caused by postural, vestibular, neurological and muscular aspects, and falls are one of the most severe outcomes in the elderly. More than 1/3 of adults fall each year; in particular, one up to 10 falls result in serious injury, with 40% of injury-related deaths in adults over age 65 (60).

## Benefit of exercise

Regular physical activity is crucial for maintaining good health, as is the case with older people, and it also promotes positive mental health. It can also reduce the risk of moderate or severe functional limitations and the risk of premature death in older adults (15, 61). The advantages of physical exercise and physical activity are evident for both healthy subjects and patients (62). Different data showed that physical exercise and physical activity determines an improvement of the physical form, defined as a state of well-being characterized by a low risk of incurring premature health problems and energy to spend on various activities. Notably, in some prospective observational studies, including type 2 diabetes mellitus, thromboembolic stroke, hypertension, osteoporosis, obesity, colon cancer, breast cancer, anxiety and depression, regular physical activity is inversely related to disease outcomes (43) (Table 2).

**Table 2:** Summary of the benefit of exercise.

Exercise benefits
Cardiovascular and pulmonary systems
Increased heart rate variability. Better endothelial reactivity. Lower inflammatory markers. Reduced arterial stiffness, improved cardiac output. Less atherosclerotic disease; enhanced microvasculature. Improved gas exchange. Stronger respiratory muscle. Improves muscle function and adaptation to oxidative stress. Improve the function of mitochondria in the skeletal muscle. Improves vascularization. Reduces cardiovascular disease risk. Reduces the risk of heart failure. Reduces the risk of heart attack. Reduces the risk of peripheral vascular disease. Reduces high blood pressure. Lowers LDL and total cholesterol. Reduces the risk of stroke
Musculoskeletal
Increases muscle mass, strength, and power. Preserves and increases bone mass. Improves and maintains joint range of movement and flexibility. Increases synthesis of collagen in ligaments and tendons. Reduces the risk of osteoporosis and risk of osteoporotic fracture, especially among postmenopausal women. Reduces the risk of sarcopenia and frailty syndrome. Reduces age-related loss of muscle mass and improves muscle mass and strength, and physical function.
Neurologic and neuropsychological systems
Faster nerve conduction. Improved balance. Improved memory, attention and reaction time by increasing the size of the hippocampus and cortical volumes. Improved visual-spatial orientation and proprioception. Improved sleep. Reduces the risk of dementia. Prevents mild cognitive decline. Improves memory function. Physical inactivity is among the top reversible risk factors for Alzheimer's disease (which also includes hypertension, obesity and diabetes).
Respiratory system
Improved gaseous exchange. Increased vital capacity. Increased tidal volume. Improved O2 debt tolerance. Lungs work more efficiently. Increase oxygen to the body. Increase VO_2_ max.
Immune and endocrine systems
Reduce markers of systemic inflammation. Increased basal metabolic rate. Improved lipid profiles. Lower body fat percentage. Improved insulin sensitivity and glucose homeostasis. Reduces the risk of obesity-related conditions. Reduces risk of diabetes mellitus.

## Exercise prescription: frequency, intensity, type and time

The term “dose” can have multiple interpretations when used in descriptions of physical activity. It can be referred to as total energy expended, particularly the intensity, duration, or frequency of activity (63, 64). Exercise prescription is based on a specific format: FITT (frequency, intensity, type, and time) (2). Frequency refers to the number of times an activity is performed per week; the intensity is defined as the level of vigour at which the exercise is performed; the time refers to the length of time that the activity is performed and typically indicates the metabolism involved during the exercise (Supplementary Tables S1, S2).

In order to maintain good health, healthy adults aged 18–65 should engage in moderate-intensity aerobic physical activity for at least 30 min on five days each week. Alternatively, they can do vigorous-intensity aerobic exercise for at least 20 min three days each week. Combining moderate and vigorous intensity activities is also an option to meet this recommendation. Additionally, to promote physical independence and maintain good health, adults should engage in activities that support or increase muscular strength and endurance for at least two days each week. To effectively develop strength, it is recommended to use resistance (weight) that allows for 8–12 repetitions of each exercise, leading to volitional fatigue. Activities that aid muscle-strengthening include a progressive weight-training program, weight-bearing callisthenics, stair climbing, and other resistance exercises that engage major muscle groups. The American College of Sports Medicine recommends exercise prescription for healthy adults. The Supplementary Tables 1S–S3 suggested for developing and maintaining cardiorespiratory fitness, body composition, muscular strength and endurance, and flexibility for healthy adults.

## New health care approach in older people: a multidimensional approach

Currently, healthcare professionals are experiencing a demographic shift, with an increase in the elderly population. who have multiple chronic illnesses and are more complex to treat than younger individuals with single-organ damage. The growing demand for assistance from this share of the population with a prevalence of chronic diseases results in a significant economic impact on the health care system. Moreover, ageing requires integrated, efficient and effective management in which the patient and the relative caregiver must be active protagonists. In this sense, the single specialist approach, which considers a single condition, must be transformed into teamwork, considering people with comorbidities as their whole at the centre of clinical care. According to this vision, the case management approach, including nursing coordination, nutritional assessment and support, and physical exercise and physical activity capability assessment, seems appropriate. The multidimensional approach should limit a surge in resource consumption (increase in health expenditure) and is based on the professional interaction between local health services, specialists, and other health care providers.

The specific objectives of the multidimensional model are how to classify the patient according to categories of complexity (35), how to evaluate the elderly patient's complexity according to a vision that takes into consideration comorbidities and frailty including sarcopenia and nutritional deficiency, physical, metabolic, cardiovascular and respiratory impairments, how to share projects, paths and contents with patient's associations, how to evaluate residual functional capacity and how to plan a customized and monitored motor training program, how to develop an integrated management program hospital-territory assistance services. In this sense, older people with diabetes mellitus, with their constellation of cardiac, metabolic, neurological and renal complications, represent the typical complex patient. The management of these patients represents a shift from a reactive strategy to a proactive approach, meant as a comprehensive approach that covers all aspects of their health. The integration of territorial, hospital and social assistance services defines the thoroughness of the management (64).

As previously described by Nardi et al. (35), four categories of complexity are possible: Know, Knowable, Complex and Chaotic. The local services must manage the first two. The other two tertiary care hospitals are linked to territorial health care services and professionally tailored follow-up.

In particular, the multidimensional model provides for an evaluation of clinical data using the Cumulative Illness Rating Scale or comorbidity index (CIRS) (65, 66), the Barthel Index (66, 67), and the Geriatric Depression Scale (GDS) (67, 68). The CIRS allows the investigation of the individual's state of health by defining the clinical and functional severity of 14 categories of diseases. The scale provides a score from 0 to 56 (the higher the score, the worse the patient's state of health). Subsequently, each patient completes a cardiovascular, respiratory, metabolic and motor evaluation. In addition, the patient's cognitive sphere is also evaluated through the analysis of the areas: depression, guilt, suicidal ideas, initial insomnia, intermediate insomnia, prolonged insomnia, work and interests, slowing of thought and words, agitation, the anxiety of psychic origin, the anxiety of somatic origin, gastrointestinal somatic symptoms, somatic symptoms general, genital symptoms, hypochondria, introspection, weight loss, daily change in symptoms, depersonalization, paranoid symptoms, obsessive symptomatology.

Moreover, the Barthel Index evaluates the autonomy in daily life activities by the analysis of 10 different items that describe the activities of daily life, such as the capacity to feed, dress, manage personal hygiene, the ability to control the bone and urination, wash, move from chair to bed, walking level, go up and down the stairs. After the patient's discharge, one detailed report is sent to the general practitioner, and the patient is followed through periodic telephone follow-ups. Finally, the GDS score assesses mood disorders in a form consisting of 15 items. There are ten items in a questionnaire that suggest the presence of depression if answered positively, while five other items (question numbers 1, 5, 7, 11, 13) indicate depression when answered negatively. Depending on age, education, and complaints, scores of 0–4 are considered normal. Scores of 5–8 indicate mild depression, scores of 9–11 indicate moderate depression, and scores of 12–15 indicate severe depression. The nutritional assessment, a key component of the comprehensive managementincludes waist circumference, body mass index, and the Mini Nutritional Assessment (MNA) (69). The nutritionist also performs bioimpedance analysis to assess body composition and possible sarcopenia. Calcaneal ultrasonography is used to assess bone mineral density, and a dietary regimen appropriate to the patient's clinical condition is prescribed. Patients or caregivers, receive education from a nurse about lifestyle, therapy and glycemic self-monitoring devices, and foot care from a podiatrist.

Within this model, a stratification of the risk and therapeutic choice is necessary. The presence of heart disease and or an atheromatous plaque represents the equivalent of organ damage that involves an additional CV risk, a worse prognosis and, therefore, the need for appropriate therapeutic integration and a specific nutritional and physical activity program (70). Diabetic nephropathy can be detected by measuring 24-h albuminuria, the urine albumin to creatinine ratio (ACR) and calculating the GFR (71). The importance of early diagnosis cannot be overstated, as it allows intervention to be started as soon as possible to reduce the risk of further progression of nephropathy and prevent CV complications (72).

The concept of chronic critical issues is introduced: as clinical complexity increases, the patient becomes more unstable. The critical issues can be overcome by identifying an elective access path in a specialist hospital setting in order to avoid improper emergency area admission (64).

The outcomes of this pathway should be holistic assessment with therapeutic reconciliation to control drugs, polytherapy, health care status and assessment, empowerment of the patient and his caregiver, nutritional support, physiotherapy, adapted physical activity (APA), and a tailored follow-up program.

## Conclusion

It is well known that a sedentary lifestyle contributes, along with other risk factors, to the development of many chronic-degenerative conditions, including cardiovascular, metabolic and osteoarticular diseases. Regular physical activity benefits the human body; evidence demonstrates an inverse relationship between mortality and physical activity. Therefore, it is essential for the primary prevention of many diseases. In older people, exercise produces four main benefits: it improves functional capacity both in healthy subjects and in ill patients; it supports the management of diseases such as arterial hypertension, diabetes, obesity and dyslipidaemia; it reduces the risk of developing chronic disease; it improves and reduces the cerebrovascular disease. Mainly, the benefits include (a) osteoporosis reduction; (b) the improvement of muscle tone and ability to move; (c) the decrease of the risk of sudden death due to cardiovascular diseases; (d) the induction of an increased release of neurohormonal mediators such as endorphins and serotonin; (e) the decline of the development of malignancies and metabolic diseases; and (f) the slow-down of the decline of cognitive skills. Although the beneficial effects of exercise increase with the rising frequency of the intensity of the activity, it is essential to remember that the most significant benefits are related to moderate-type physics activity.

A complete program that integrates aerobics and anaerobic activities finds a safer indication in the elderly subject without confirmation pathology than in frail elderly subjects at high risk of disability. The physical activity must be adequate to the subject's abilities (organic, psychic, functional, etc.). In particular, it must be prescribed after an accurate evaluation of the people's conditions, according to environmental aspects and established intensity, duration and rhythm of the exercises. The exercise must be a global stimulus for the whole body and not only for some sectors' systems and equipment; it must be agreeable and suitable for the cultural, creative and effective possibilities of the subject being part of a global lifestyle program. According to what has been said above, field research would be desirable to verify the effectiveness and efficiency of this approach.
