# Corrigendum: Ageing, clinical complexity, and exercise therapy: a multidimensional approach

**DOI:** 10.3389/fspor.2025.1602307

**Published:** 2025-04-09

**Authors:** Salvatore Corrao, Dario Cerasola, Daniela Lucini, Christiano Argano

**Affiliations:** ^1^ Department of Health Promotion Sciences, Maternal and Infant Care, Internal Medicine and Medical Specialties, (PROMISE), University of Palermo, Palermo, Italy; ^2^ Department of Internal Medicine, National Relevance and High Specialization Hospital Trust, ARNAS Civico, Di Cristina, Benfratelli, Palermo, Italy; ^3^ Department of Psychology, Educational Science and Human Movement, University of Palermo, Palermo, Italy; ^4^ Department of Experimental Biomedicine and Clinical Neurosciences (BioNeC), University of Palermo, Palermo, Italy; ^5^ BIOMETRA Department, University of Milan, Milan, Italy; ^6^ Exercise Medicine Unit, Istituto Auxologico Italiano, IRCCS, Milan, Italy

**Keywords:** ageing, clinical complexity, exercise therapy, physical exercise, active ageing, multidimensional approach

A Corrigendum on Ageing, clinical complexity, and exercise therapy: a multidimensional approach By Corrao S, Cerasola D, Lucini D and Argano C (2025). Front. Sports Act. Living 6:1422222. doi: 10.3389/fspor.2024.1422222

In the published article, there was a mistake in the Funding statement. The following funding statement was reported incorrectly: “The author(s) declare that no financial support was received for the research, authorship, and/or publication of this article”.

The correct funding statement appears below.

FUNDING

“This work was supported by the Italian Ministry of Health and by Regione Sicilia (project ID NET-2018-12367032-4)”.

The authors apologize for this error and state that this does not change the scientific conclusions of the article in any way. The original article has been updated.

