# Supplementary material for: Ageing, clinical complexity, and exercise therapy: a multidimensional approach
Source: Front Sports Act Living. 2025 Jan 6;6:1422222. doi: 10.3389/fspor.2024.1422222 (PMC11743540; doi:10.3389/fspor.2024.1422222)
Supplement: Supplementary file 1 [file Table1.docx]

**Supplement table 1.** Measure exercise intensity recommended by ACSM

| **Exercise Intensity** | | | | | |
| --- | --- | --- | --- | --- | --- |
|  | Personal evaluation | | Physiological measures | |  |
| Intensity | Talk Test | BORG  (10 point scale) | %HRR-%VO2 | Maximal HR (%) | METs |
| Light | Able to talk/sing | ≤3 | ≤40 | ≤64 | ≤3 |
| Moderate | Able to talk but not sing | 4-5 | 40-60 | 65-76 | 4-6 |
| Vigorous | Difficulty talking | ≥6 | ≥61 | ≥77 | ≥7 |
