# Supplementary material for: Ageing, clinical complexity, and exercise therapy: a multidimensional approach
Source: Front Sports Act Living. 2025 Jan 6;6:1422222. doi: 10.3389/fspor.2024.1422222 (PMC11743540; doi:10.3389/fspor.2024.1422222)
Supplement: Supplementary file 2 [file Table2.docx]

**Supplement table 2.** Intensity Classification based on physical activity lasting up to 60 min

| **Relationship between exercise intensity and type of actitity** | | | | | | | | |
| --- | --- | --- | --- | --- | --- | --- | --- | --- |
|  | Resistance exercise | Endurance Exercise | | Exercise Intensity relate to age | | | |  |
| Intensity | Maximal Contraction (%) | VO_2_max (%) | Maximal heart Rate (%) | Young (≤39 yrs) (METs) | Middle-aged (40/64 yrs) (METs) | Old (65/79 yrs) (METs) | Very Old (≥80 yrs) (METs) | Borg scale (0-20) |
|  |  |  |  |  |  |  |  |  |
| Light | ≤50 | 20-40 | 35-55 | 2.4-4.7 | 2.0-3.9 | 1.6-3.1 | 1.1-1.9 | 10/11 |
| Moderate | 51/70 | 41-60 | 56-70 | 4.8-7.1 | 4.0-5.9 | 3.2-4.7 | 2.0-2.9 | 12/13 |
| Hard | 71/85 | 61-85 | 71-90 | 7.2-10.1 | 6.0-8.4 | 4.8-6.7 | 3.0-4.25 | 14/16 |
| Very Hard | ≥86 | ≥86 | ≥91 | ≥10.2 | ≥8.5 | ≥6.8 | ≥4.3 | 17/19 |
