# Supplementary material for: Ageing, clinical complexity, and exercise therapy: a multidimensional approach
Source: Front Sports Act Living. 2025 Jan 6;6:1422222. doi: 10.3389/fspor.2024.1422222 (PMC11743540; doi:10.3389/fspor.2024.1422222)
Supplement: Supplementary file 3 [file Table3.docx]

**Supplement table 3**. Summary of ACSM/AHA physical activity recommendations for older adults.

| **Physical activity recommendations for older adults** |
| --- |
| Endurance exercise  Frequency: For moderate-intensity activities, accumulate at least 30 or up to 60 (for greater benefit) min in bouts of at least 10 min each to total 150–300 min , at least 20–30 min or more of vigorous-intensity activities to total 75–150 min , an equivalent combination of moderate and vigorous activity.  Intensity: On a scale of 0 to 10 for the level of physical exertion, 5 to 6 for moderate-intensity and 7 to 8 for vigorous intensity.  Duration: For moderate-intensity activities, accumulate at least 30 min in bouts of at least 10 min each or at least 20 minIdj1 of continuous activity for vigorous-intensity activities.  Type: Any modality that does not impose excessive orthopedic stress; walking is the most common type of activity. Aquatic exercise and stationary cycle exercise may be advantageous for those with limited tolerance for weight-bearing activity.  Resistance exercise: Frequency: At least 2 dI.  Intensity: Between moderate- (5–6) and vigorous- (7–8) intensity on a scale of 0 to 10.  Type: Progressive weight training program or weight bearing callisthenics (8–10 exercises involving the major muscle groups of 8–12 repetitions each), stair climbing, and other strengthening activities that use the major muscle groups.  Flexibility:  Frequency: At least 2 dI.  Intensity: Moderate (5–6) intensity on a scale of 0 to 10.  Type: Any activities that maintain or increase flexibility using sustained stretches for each major muscle group and static rather than ballistic movements.  Balance: For people that have frequent fallers or mobility problems, AHA guidelines recommend balance training. While activities that include progressively difficult postures, dynamic movements and stressing postural muscle groups are recommanded by ACSM guidelines. |
